# Cytogenetic Analysis and Chromosomal Characteristics of the Polymorphic 18S rDNA of *Haliotis discus hannai* from Fujian, China

**DOI:** 10.1371/journal.pone.0113816

**Published:** 2015-02-20

**Authors:** Haishan Wang, Xuan Luo, Weiwei You, Yunwei Dong, Caihuan Ke

**Affiliations:** 1 State Key Laboratory of Marine Environmental Science, Xiamen University, Xiamen, China; 2 Colleges of Ocean and Earth Sciences, Xiamen University, Xiamen, China; Australian Museum, AUSTRALIA

## Abstract

We report on novel chromosomal characteristics of *Haliotis discus hannai* from a breeding population at Fujian, China. The karyotypes of *H. discus hannai* we obtained from an abalone farm include a common type 2n = 36 = 10M + 8SM (82%) and two rare types 2n = 36 = 11M + 7SM (14%) and 2n = 36 = 10M + 7SM + 1ST (4%). The results of silver staining showed that the NORs of *H. discus hannai* were usually located terminally on the long arms of chromosome pairs 14 and 17, NORs were also sometimes located terminally on the short arms of other chromosomes, either metacentric or submetacentric pairs. The number of Ag-nucleoli ranged from 2 to 8, and the mean number was 3.61 ± 0.93. Among the scored interphase cells, 41% had 3 detectable nucleoli and 37% had 4 nucleoli. The 18S rDNA FISH result is the first report of the location of 18S rDNA genes in *H. discus hannai*. The 18S rDNA locations were highly polymorphic in this species. Copies of the gene were observed in the terminal of long or/and short arms of submetacentric or/and metacentric chromosomes. Using FISH with probe for vertebrate-like telomeric sequences (CCCTAA)_3_ displayed positive green FITC signals at telomere regions of all analyzed chromosome types. We found about 7% of chromosomes had breaks in prophase. A special form of nucleolus not previously described from *H. discus hannai* was observed in some interphase cells. It consists of many small silver-stained nucleoli gathered together to form a larger nucleolus and may correspond to prenucleolar bodies.

## Introduction

The Pacific abalone, *Haliotis discus hannai*, which naturally inhabits temperate waters, is one of the most commercially important marine shellfish for aquaculture in eastern Asia because of its high market value and nutrient content for human consumption. In China, *H. discus hannai* is an economically important gastropod species and its large-scale cultivation started in the late 1980s [1], more than 20000 metric tons being produced annually since 2007. In recent years, the industry has performed exceptionally well in Fujian, South China, As a result, the production of abalone in Fujian accounts for about two-thirds of the total Chinese output [2].

Because of its commercial importance, several intensive studies aimed at increasing the productivity of *H. discus hannai* in culture have been carried out on, including population genetic analysis, seed production, and ecology [3–8]. With the decline of natural populations due to overfishing and environmental deterioration, abalone aquaculture is becoming more important, and genetic breeding for superior strains with fast growth rate and disease resistance has been carried out on a large scale in China over the past decade. Recent studies have shown that intraspecific crossbred abalone from geographically different abalone population stocks can produce positive heterosis in growth and survival, two of the most important commercial traits for aquaculture production. In China, selective crossbreeding techniques have been applied extensively in production because of the demand for genetic improvement in abalone lines. For aquaculture and breeding of abalone, a wide range of biological knowledge is required. However, whilst some fundamental biological studies have been conducted on *H. discus hannai*, modern cytogenetic studies, which are important with regard to the genetics and breeding science of the species, are scarce.

The application of the fluorescence *in situ* hybridization (FISH) technique, which enables visualization of target DNA sites on chromosomes through a signal display using probes (gene mapping), is useful for future research on *Haliotis* species. In reference to abalones, although there have been many chromosomal studies on *Haliotis*, most of them presented only basic karyological data for species such as the number and structure of the chromosomes present [9–15]. To date there are only a few available karyological studies using FISH, such as location of ribosomal DNA(rDNA) in *H. rufescens*, analysis of telomeres and investigating the presence of (GATA)n microsatellites[16–19].

The karyotype of *H. discus hannai* has been reported as 2n = 36. However, there are differences between reports with respect to the number of metacentric and submetacentric chromosome pairs found (10M + 8SM and 11M + 7SM respectively) [10,15,20]. A silver staining study on *H. discus hannai* indicated that the NORs were generally located terminally on the long arms of two chromosome pairs, although there are cvariations in the NOR-bearing chromosome[10]. The research of Sakai et al suggested that the telomere of *H. discus hannai* is composed of a (TTAGGG)n sequence[18]. The rDNA location of the Pacific abalone *H. discus hannai* has not been reported. Therefore, we attempted to determine the location of the rDNA using the FISH technique.

We used silver-staining and FISH with a 18S rDNA probe to investigate the location of rDNA in *H. discus hannai*. We also used FISH, telomere PNA probes with vertebrate-like telomeric sequences (CCCTAA)_3_ labeled with Cy3 to examine the distribution of telomeric DNA in *H. discus hannai*.

## Materials and Methods

### Abalone Collection

*H. discus hannai* were obtained from Hongyun Abalone hatchery in Fujian Province. This abalone from the north coast of China is widely naturalized on the Fujian coast and has been artificially propagated for several generations. As *H. discus hannai* is not a protected species, and collections were only made from public access areas, no specific permits were required to collect this species from these locations/activities.

### Chromosome Preparation

Specimens for chromosomal studies were obtained according to the conventional method [20] with minor modifications. The trochophores were collected and cultured in 0.03% colchicine at room temperature (~20°C) for 1 hour. The larvae were then exposed to 0.075 M KCl solution for 45 min, then fixed three times (30 min each) with Carnoy’s fixative (ethanol: glacial acetic acid = 3:1), and stored at -20°C until use. The fixed larvae were dissociated into fine pieces by gentle pipetting in 50% acetic acid solution and the resulting cell suspension was dropped onto a preheated glass slide and air dried. The chromosome preparations for FISH were preserved at -20°C until use.

### Karyotyping and Ag-staining of the NORs

The NORs in metaphase and the nucleoli in interphase were detected using silver nitrate staining with minor modifications; the silver colloidal solution was 1 part by volume of 2% gelatin in 1% formic acid and two parts by volume of 20% aqueous silver nitrate solution. The slides were stained in a closed Coplin jar for 20 min at a temperature of 37°C. The silver colloidal solution was washed with double distilled de-ionized water. The karyotype was established from the conventional Ag-NORs stained metaphase, and the length of the short and long arms and total chromosome length were measured with Image-Pro Plus 6.0 software (Media Cybernetics, USA). The relative lengths (percentage of the total length of all chromosomes) and arm ratio (ratio of long arm to short arm lengths) for each chromosome were determined from these values. Nomenclature for the centromeric position on the chromosome was according to Levan [21] based on the arm index (metacentric chromosomes, 1.0–1.7; submetacentric, 1.7–3.0; subtelocentric, 3.0–7.0; telocentric, 7.0-N). Positioning of *H. discus hannai* chromosomes within the karyotype was calculated based on relative chromosome length and arm index. The karyotype was constructed by first dividing the chromosome pairs into classes on the basis of centromere position, and then arranging the homologous pairs in order of decreasing length within each group.

### Fluorescence *In situ* Hybridization

The probe for detecting 18S rDNA was labeled with Biotin-16-dUTP (Roche Germany) using PCR amplification with primers developed for this manuscript: 18SF: 5’-CTTCCGTCAATTCCTTTAAGTT-3’, 18SR: 5’-TCTATCAAGTGTCTGCCCTATC-3’ using genomic DNA from *H. discus hannai* as the template. After obtaining the labeled probe, a hybridization solution was added at 80°C for 7 min to denature the DNA probe and chromosomal DNA. FISH was performed at 37°C overnight. After hybridization, the slides were washed twice in 50% formamide/2×SSC for 5 min at 40°C, and 2×SSC and 1×SSC for 5 min at RT. Biotinylated probes were detected with avidin-FITC (Invitrogen, USA). Chromosomes were counterstained with PI (1.5 ug/mL Vector) at room temperature for 10 min. FISH using vertebrate telomere PNA probes with Telomere PNA FISH KIT (PANAGE, Korea) was performed according to the manufacturer’s instructions. Briefly, the slide was placed in a preheated incubator at 80°C for 5min, 20 μl of PNA probe in Hybridization buffer was added to the marked area on each slide. The marked area on each slide was covered with an 18x18 mm cover slip, and the slide was denatured for 10 min at 85°C. The slide was immersed in Washing solution (2×SSC/0.1% TWEEN-20) at RT to remove the coverslips. Then the slide was washed in Washing solution for 10 min at 56°C twice, and 1 min at RT. The slide was stained for 10 min in the DAPI/2XSSC solution, and 2×SSC for 2 min, 1×SSC for 2 min, twice in distilled water for 2 min. Then it was covered with a coverslip and the solution was allowed to spread evenly under the coverslip. The exposed slide was visualized using an Olympus epifluorescence microscope (BX51, Japan) equipped with a CCD camera and analyzed with Image-Pro Plus 6.0 software (Media Cybernetics, USA).

## Results

### Karyotyping and Ag staining of the NORs

**Fig 1:**
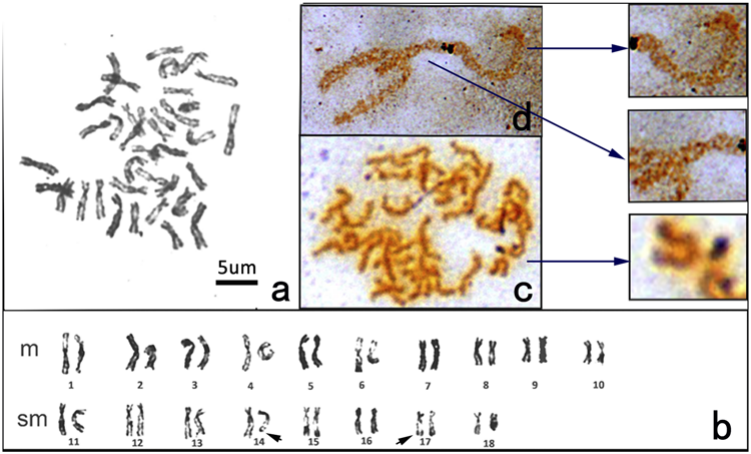
Karyotypes and NOR-bearing chromosomes of *H. discus hannai*. a: Digital image of metaphase plate from larvae of *H. discus hannai*. b: Karyotype of *H. discus hannai*. c: A metaphase of *H. discus hannai* with a short arm terminally NOR-bearing chromosome. d: A metacentric chromosome with short arm terminally NOR-bearing and a submetacentric chromosome with its short arm terminally NOR-bearing. (The arrows indicate zoom from chromosomes from parts c and b.)

A total 50 metaphases examined from *H. discus hannai* showed a diploid complement of 2n = 36. The karyotypes of 41 of these (82%) consisted of 10 metacentric pairs and eight submetacentric pairs (Fig. 1a&b). Two rare karyotypes were also observed, one with 2n = 36 = 11M + 7SM (14%) and one with 2n = 36 = 10M + 7SM + 1ST(4%). Based on chromosome relative lengths (“RL”) and arm ratio from the trochophore larvae, in *H. discus hannai* the maximum chromosome RL was 7.41 ± 0.16 and the minimum was 3.91 ± 0.17 (Table 1). It is worthwhile to note that the relative length of the fifth chromosome pair was 6.39 ± 0.42 and arm ratio was 1.45 ± 0.31, the No. 17 chromosome pair RL = 4.21 ± 0.45 and AR = 2.94 ± 0.24. Both the fifth pair and the seventeenth pair have a large standard deviation. In all metaphases we observed the AR of some fifth pair chromosomes was greater than 1.7, making it a submetacentric chromosome pair under our defnition. The NORs were usually located terminally on the long arms of 14 and 17 chromosome pairs (Fig. 1b). However, many variations in the NOR-bearing chromosomes were observed, the variation occurring in both the number of NORs and/or their specific locations. The NORs in some individuals were also located terminally on the short arms of chromosomes other than pairs 14 and 17, including both metacentric and submetacentric pairs. The chromosome numbers of each pair bearing the NOR in other metaphases were identified by referring to the relative chromosome lengths and arm ratios illustrated in Fig. 1 c&d. The other chromosome pairs bearing the NOR were identified as numbers 4, 9 and 13.

**Table 1:** Relative lengths and arm ratios of chromosomes of *H. discus hannai*.

No.	Relative length Mean± SD	Arm ratio Mean± SD	Type
1	7.41 ± 0.16	1.34 ± 0.05	m
2	7.14 ± 0.27	1.20 ± 0.07	m
3	6.88 ± 0.20	1.21 ± 0.07	m
4	6.67 ± 0.31	1.22 ± 0.08	m
5	6.39 ± 0.42	1.45 ± 0.31	m/sm
6	6.18 ± 0.23	1.16 ± 0.08	m
7	5.67 ± 0.25	1.51 ± 0.16	m
8	4.64 ± 0.29	1.38 ± 0.24	m
9	4.32 ± 0.38	1.22 ± 0.21	m
10	4.25 ± 0.28	1.24 ± 0.14	m
11	6.31 ± 0.33	1.82 ± 0.15	sm
12	5.92 ± 0.28	2.32 ± 0.27	sm
13	5.69 ± 0.13	2.82 ± 0.30	sm
14	5.65 ± 0.14	2.63 ± 0.37	sm
15	5.26 ± 0.26	2.50 ± 0.21	sm
16	4.50 ± 0.29	2.13 ± 0.39	sm
17	4.21 ± 0.45	2.94 ± 0.24	sm/st
18	3.91 ± 0.17	2.20 ± 0.31	sm

m: metacentric. sm: submetacentric. st: submetacentric.

### FISH with 18S Probe and telomere sequence

**Fig 2:**
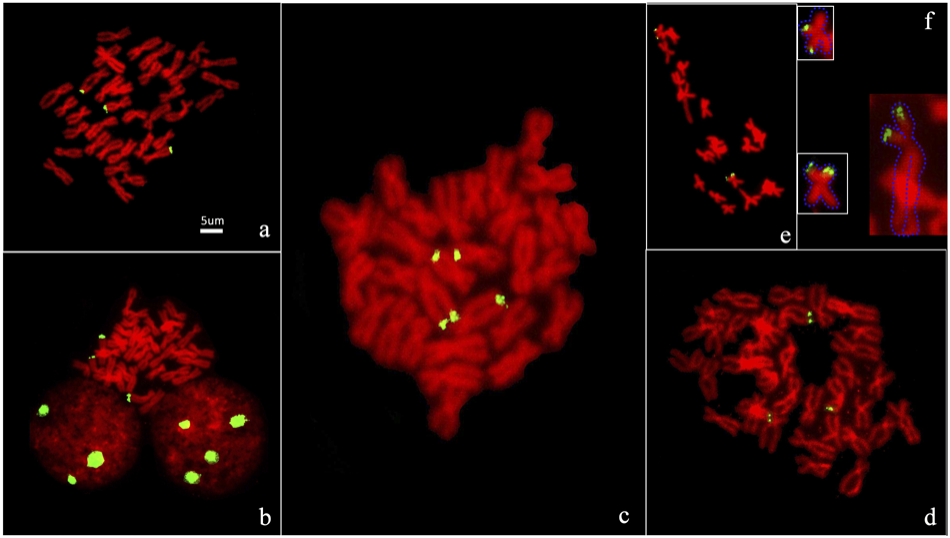
18S rDNA location on chromosome of *H. discus hannai*. a: Three rDNA signals on *H. discus hannai* chromosomes. b: 3 rDNA signals on chomosomes and signals on interphase cells with 3 and 4 nucleoli. c: The common type of rDNA location on chromosomes pairs 14 and 17. d: The rare type of rDNA location on chromosomes short arm in metaphase. e&f: strong signals terminally on the short arms of both m and sm chromosomes. (FITC shown as yellow/green color, PI shown as red color and the blue dashed line in order to better display the outline of chromosome diagrammatically.

In the majority of the 93 individual larval metaphases that we scored, hybridization of the 18S ribosomal probe revealed multiple 18S ribosomal gene clusters located on three or four chromosomes (Fig. 2). The 18S-rDNA probe produced strong signals on the telomere of the long arms of submetacentric chromosome pairs 14 and 17 in 81 (87%) metaphases (Fig. 2c). However some different rDNA patterns (13%) were observed. Most of these different patterns were found in rare karyotypes. For example a signal was detected on a subtelocentric chromosomes(Fig. 2f), it belongs to an individual with 2n = 36 = 10M + 7SM + 1ST. More details about the ariant patterns follow. (1) The rDNA was located near the telomere of the long arms in three non-homologous chromosomes: two submetacentric and one metacentric chromosome (Fig. 2a). (2) the rDNA was located on three different submetacentric chromosomes (Fig. 2b). (3) 18S signals occurred on the telomere of the long arms of one submetacentric chromosome and on the telomere of the short arm of one pair of metacentric chromosomes (Fig. 2d). (4) The 18S rDNA probes can be detected on the telomere of long or/and short arm chromosomes and subtelocentric or/and metacentric chromosomes (Fig. 2e&f). In addition, the specific localization of regions with low intensity showed a high cytogenetic variability in all the analyzed metaphases.

**Fig 3:**
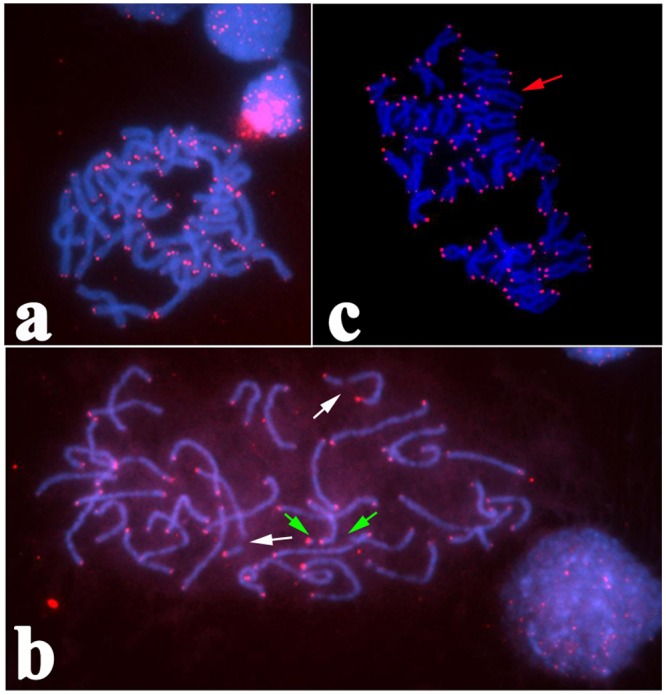
18S rDNA location on chromosome of *H. discus hannai*. a: Mitotic metaphase chromosomes after FISH treatment with the telomeric sequence (CCCTAA)_n_. b: Three breaks on prophase chromosome of *H. discus hannai*, white arrows indicate two breaks in the centromere region and the green arrow indicates the terminal break. c: A rare karyotype with st chromosome, red arrow indicated st chromosome. (cy_3_ shown as red color and DAPI shown as blue color.)

FISH with the PNA and (CCCTAA)_3_ oligonucleotide probes displayed clearly defined signals on the telomeric regions of all metaphase chromosomes and interphase nuclei. These results suggest that the telomere of *H. discus hannai* is composed of a (CCCTAA)n sequence, which is typically found in vertebrates and other abalone[16,18]. FISH using the vertebrate telomere PNA probe displayed positive red Cy_3_ signals at telomere regions of all analyzed chromosome types (Fig. 3a). The visualization of the interphase nucleus labelled with a telomere probe provided evidence of the distribution of telomeres at the nuclear periphery (Fig. 3a). We found about 7% of the karyotypes had a chromosomal breaks in prophase. For example in one cell, two breaks occurred between the telomere region and centromere position, one break on the telomere region (Fig. 3b). An unusual karyotype with two subtelocentric chromosomes was occurred, and this metaphase had only 34 chromosomes instead of 36 (Fig. 3c).

### Ag-nucleoli and 18S gene of interphase cells

**Fig 4:**
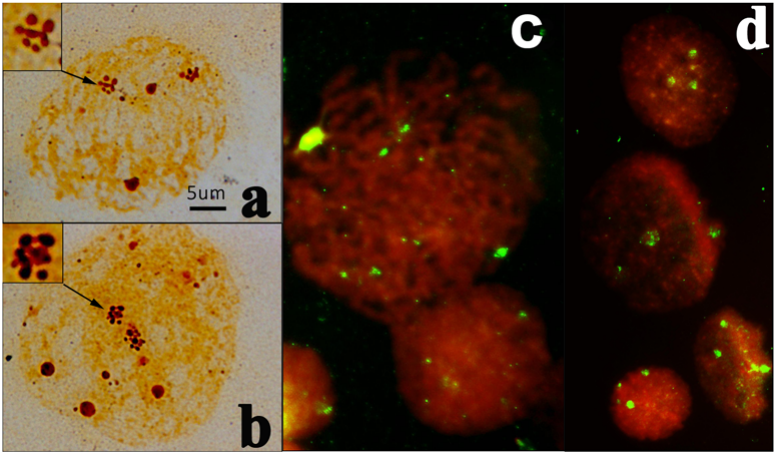
Ag-nucleoli and 18S gene of interphase cells. a: 4 NORs in prophase with 2 normal NORs and 2 conglomerate NORs composed of many small NORs. b: 5 NORs on metaphase with 2 NORs composed with of small NORs. c: 18S rDNA on prophase cell after FISH. d: FISH with 18S gene on metaphase cell.

Ag-nucleoli in interphase cells, like NORs in metaphase cells, can be visualized by silver staining. A total of 87 interphase cells of *H. discus hannai* were analysed. The number of Ag-nucleoli ranged from 2 to 8, and the mean number of 3.61 ± 0.93 was similar to that of the Ag-NORs observed in metaphases. Among the scored interphases 41% had 3 nucleoli and 37% had 4 nucleoli. There was a special form of nucleolus not previously described from this species. In 2.9% of interphase cells many small silver stain nucleoli were gathered together to form a larger nucleolar-like structure (Fig. 4a&b). There were only one or two structures of this form in any one interphase cell and other nucleoli in these cells had a normal appearance;. In contrast, FISH results indicated 4 strong signals in 78% of interphase cell and 3 signals in 22% (Fig. 4d). There was some other faint fluorescence also detected in interphase cells, especially in prophase chromosomes (Fig. 4c).

## Discussion

The chromosome number of *H. discus hannai* found in the present study was 2n = 36. The karyotype of larvae we collected from an abalone farm in Fujian had a common type 2n = 36 = 10M + 8SM (82%) and two rare types 2n = 36 = 11M + 7SM (14%) and 2n = 36 = 10M + 7SM + 1ST (4%). Other studies have reported the same common karyotype for *H. discus hannai*[10]. However, there are differences between reports with respect to the number of metacentric and submetacentric chromosome pairs found (e.g. 11M + 7SM) [15,20]. Chromosome polymorphism in shellfish is a common phenomenon[22]. The AR of the fifth and the seventeenth chromosome pairs have a large standard deviation. The AR of some fifth pair chromosomes was greater than 1.7 and the AR of some representatives of the seventeenth pair was greater than 3.0. Possibly, structural changes associated with these two chromosome pairs cause the uncommon karyotype.

The Ag-NOR has been a useful chromosome marker, showing variations in such properties as number, location and size, that are often species specific. With regards to abalone, NOR-bearing regions have only been reported in *H. discus hannai* by silver staining. According to Okumura[10], NORs were terminally located on the long arms of two chromosome pairs. However, the specific localization was shown to be variable, with NORs found among submetacentric chromosomes as well as metacentric chromosomes. In the present study the NORs of *H. discus hannai* were usually located terminally on the long arms of chromosome pairs 14 and 17 (Fig. 1b). However, many variations in the number or identity of NOR-bearing chromosomes were observed. The NORs also located terminally on the short arms of chromosomes of both metacentric and submetacentric pairs. The chromosome pairs bearing the variant NORs were identified as numbers 4, 9 and 13 (Fig. 1 c&d), differing from earlier results. There is now a general consensus that many variations of NOR-bearing chromosomes are observed in *H. discus hannai*, as also found in numerous species of crustaceans, molluscs, teleosts, reptiles and mammals [23–28]. Generally speaking, intraspecific polymorphism of NOR could be supposed to be due to the fact that Ag-NORs can only be visualized in the transcriptionally-active state: silver nitrate staining only stains those NORs which have expressed themselves during the last interphase, because silver binds to a complex of acidic protein associated with the nucleolus and nascent pre-RNA [29].

Due to the above variability of NOR location it was necessary to locate the rDNA and telomere. The 18S-rDNA FISH result was the first time the location of this gene has been reported in *H. discus hannai*. The 18S-rDNA probe produced three or four major rDNA clusters with strong signals on the telomere of the long arms of two pairs of submetacentric chromosome pairs 14 and 17 in most metaphase cells (Fig. 2c). However, highly polymorphic the location of 18S rDNA was highly polymorphic in *H. discus hannai*. The 18S rDNA probes can be seen in the terminal of long or/and short arm chromosomes and submetacentric or/and metacentric chromosomes (Fig. 2). Variation in the number of risobosomal DNA clusters is unusual but also appears in the red abalone, according to Gallardo-Escarate [16], whose results from rDNA-FISH in *Haliotis rufescens* suggest that the copy number of rDNA is variable and may derive from mistakes of either deleting or duplicating rDNA clusters during the replication state.

The extreme environmental conditions, in terms of salinity, O_2_ concentration and water temperature, to which artificial propagation exposes *H. discus hannai* in subtropical hatcheries might contribute to the promotion of the observed inter-individual diversity in the location of rDNA genes. In fact, it has been demonstrated that adverse environmental conditions would favour chromosome breakage, mainly in correspondence with highly repeated DNA regions including NORs, due to the extreme fragility of these sites [30]. The specimens we examined came from the hatchery after several generations of inbreeding, and this may have caused high frequency of abnormal karyotypes. It is thus possible that the traditional breeding methods are restricting the development of the abalone industry.

There was a special form of nucleolus observed here that has not been described before from *H. discus hannai*. In some interphase many small silver-stained nucleoli gathered together to form a larger structure (Fig. 4a&b). The new type of nucleolus appears to corresepond to the prenucleolar bodies (PNBs) described from other species as they have a similar structure and are distributed around the chromosomes [31–33]. In some species, in diploid cells, there is one pair of NOR-bearing chromosomes, and one nucleolus is generated around each NOR. In other species, there are several pairs of NORs, and in this case several NORs participate in the building of one nucleolus. There are several models that explain how several chromosome territories contribute to one functional nuclear domain[34,35]. We hypothesize that nucleolar assembly in *H. discus hannai* depends not only on the major rDNA cluster but also on minor rDNA clusters from different chromosomes. This is in agreement with the results of previous studies[36]. This hypothesis might explain the contradiction between high polymorphism observed with 2–8 NORs in different cell and each cell can maintain the main NOR function on the early stages of larva development.
